# AMPK activates Parkin independent autophagy and improves post sepsis immune defense against secondary bacterial lung infections

**DOI:** 10.1038/s41598-021-90573-0

**Published:** 2021-06-11

**Authors:** Nathaniel B. Bone, Eugene J. Becker, Maroof Husain, Shaoning Jiang, Anna A. Zmijewska, Dae-Won Park, Balu Chacko, Victor Darley-Usmar, Murielle Grégoire, Jean-Marc Tadie, Victor J. Thannickal, Jaroslaw W. Zmijewski

**Affiliations:** 1 Department of Medicine, University of Alabama at Birmingham, Birmingham, AL 35294-0012 USA; 2 Department of Nephrology, University of Alabama at Birmingham, Birmingham, AL 35294-0012 USA; 3 Department of Pathology, University of Alabama at Birmingham, Birmingham, AL 35294-0012 USA; 4 INSERM, EFS Bretagne, UMR U1236, Université Rennes, Rennes, France; 5 Pulmonary, Allergy & Critical Care Medicine, School of Medicine, University of Alabama at Birmingham, 901 19th St. South, BMRII 406, Birmingham, AL 35294 USA

**Keywords:** Antimicrobial responses, Autophagy

## Abstract

Metabolic and bioenergetic plasticity of immune cells is essential for optimal responses to bacterial infections. AMPK and Parkin ubiquitin ligase are known to regulate mitochondrial quality control mitophagy that prevents unwanted inflammatory responses. However, it is not known if this evolutionarily conserved mechanism has been coopted by the host immune defense to eradicate bacterial pathogens and influence post-sepsis immunosuppression. Parkin, AMPK levels, and the effects of AMPK activators were investigated in human leukocytes from sepsis survivors as well as wild type and *Park2*^−/−^ murine macrophages. In vivo, the impact of AMPK and Parkin was determined in mice subjected to polymicrobial intra-abdominal sepsis and secondary lung bacterial infections. Mice were treated with metformin during established immunosuppression. We showed that bacteria and mitochondria share mechanisms of autophagic killing/clearance triggered by sentinel events that involve depolarization of mitochondria and recruitment of Parkin in macrophages. Parkin-deficient mice/macrophages fail to form phagolysosomes and kill bacteria. This impairment of host defense is seen in the context of sepsis-induced immunosuppression with decreased levels of Parkin. AMPK activators, including metformin, stimulate Parkin-independent autophagy and bacterial killing in leukocytes from post-shock patients and in lungs of sepsis-immunosuppressed mice. Our results support a dual role of Parkin and AMPK in the clearance of dysfunctional mitochondria and killing of pathogenic bacteria, and explain the immunosuppressive phenotype associated Parkin and AMPK deficiency. AMPK activation appeared to be a crucial therapeutic target for the macrophage immunosuppressive phenotype and to reduce severity of secondary bacterial lung infections and respiratory failure.

## Introduction

Severe infections, trauma, hemorrhage and burns are significant health and economic burdens with the highest in-hospital mortality rates among critically ill patients^1,2^. These conditions are often associated with development of life threatening sepsis characterized by cytokine storm, defective immune cell migration, diminished microbial clearance and multiple organ injury^3,4^. It is important to note that mortality rates are even higher due to post-sepsis complications. In particular, the diminished capacity for immune cells to eradicate pathogens persists among sepsis survivors and is linked to high risk of secondary bacterial lung infections and respiratory failure^5–7^. More than 500,000 sepsis survivors in the United States each year are at increased risk of lung infections and sepsis-related lung injury^5,8^, for which limited therapeutic interventions exist^9,10^. Aging and age-related diseases such as Parkinson’s disease (PD) are also associated with a higher risk of developing lung infections^11^, although mechanisms are not completely understood. Parkin is an E3 ubiquitin ligase that catalyzes addition of ubiquitin moieties to mitochondrial outer membrane proteins, thereby tagging depolarized mitochondria for degradation via mitophagy^12,13^. Although Parkin dysfunction has been implicated in accumulation of defective mitochondria and development of PD, recent studies indicate that loss of mitochondrial-Parkin signaling may contribute to cancer and chronic pulmonary diseases^14,15^. Leukocytes from individuals with PD exhibit many abnormalities, which may contribute to the inability to recover from exacerbations of PD after systemic infections^16,17^. Patients with PD have high mortality rates (60%), largely due to high incidence of aspiration pneumonia^18^. The poor clinical outcomes of human subjects with PD are not well understood and may relate to a fundamental deficiency in host defense functions.

It is not known whether autophagy/mitophagy evolved as a fundamental mechanism for mitochondrial quality control to maintain cellular bioenergetics or for host defense against pathogens, although both are essential for survival in metazoans^19^. Interestingly, it has been postulated that mitochondria originated from endosymbiosis of bacteria that favored enhanced bioenergetic capacity and plasticity of eukaryotic cells^20^. AMP-activated protein kinase (AMPK) is a master bioenergetic sensor and metabolic regulator, including mitochondrial function and starvation-related activation of autophagy^21^. When oxygen and nutrient bioavailability is limited, AMPK activation lowers energy expenditure and promotes a shift from anabolic to catabolic pathways, including autophagy. AMPK stimulates autophagy by direct phosphorylation of Beclin-1 and ULK1, or indirectly, via inhibition of the mTOR signaling pathway^22–24^. Although Parkin and AMPK are major activators of autophagy/mitophagy, crosstalk between these key metabolic regulators during polymicrobial sepsis and post-sepsis immunosuppression are not determined. In this report, we show acquired Parkin and AMPK deficiency in leukocytes of post-sepsis immunosuppression from human subjects and mice. We applied a mouse model of polymicrobial intra-abdominal infection to determine the effects of mitochondria-Parkin signaling axis on host defense. Next, we determined if the antidiabetic drug metformin, which induces autophagy via an AMPK-dependent mechanism, was able to enhance bacterial killing through a Parkin-independent autophagic pathway.

## Methods

### Human cells

Studies involving human blood samples were conducted in accordance with local guidelines and regulations at the Medical Intensive Care Unit, Rennes University Hospital (Rennes, France). The Université Rennes ethics committee (no 14-80/15.44-2, no 13-08) approved the study protocol and informed consent was obtained from all participants. Patients with sepsis and with the Berlin criteria for ARDS were consecutively enrolled and compared with patients who underwent bronchoscopy with normal bronchoalveolar lavage (control group of patients). Peripheral blood mononuclear cells (PBMCs) were isolated by Ficoll-Paque density gradient as previously described^25^. PBMCs were incubated in RPMI 1640 containing 7% fetal calf serum (FCS) and 1% penicillin–streptomycin, at 37 °C. After 60 min, non-adherent cells were removed by washing with complete medium. Purity of PBMC was > 80% as evaluated by flow cytometry.

### Mice

All experiments were conducted in accordance with approved protocols by the University of Alabama at Birmingham Institutional Animal Care and Use Committee. Male C57BL/6 mice and C57BL/6 *Park2*^*−/−*^ mice were purchased from The Jackson Laboratory (Bar Harbor, ME). Mice were given food and water ad libitum and kept on a 12-h light–dark cycle^26,27^. Mice 10 to 12 weeks of age were used for primary neutrophils and macrophages as well as to conduct experiments with metformin and sepsis-induced immunosuppression/secondary bacterial lung infections. The animal experiments performed for this study were carried out in compliance with the ARRIVE guidelines.

### Mouse model for sepsis-induced immunosuppression

Cecal ligation and puncture (CLP) was performed in 10- to 12-week-old male C57BL/6 mice, the current top murine polymicrobial sepsis model, according to previously established protocols implemented in our laboratory^28,29^. Briefly, the cecum was ligated approximately 25% from the cecum’s tip. A through-and-through puncture was performed with a 21-gauge needle and then a drop of feces was extruded to the peritoneal cavity. Saline (0.9%; 500 µl) was then applied into the peritoneal cavity and the abdominal wall incision was closed in two layers. The control group of mice (sham) underwent surgery without CLP. Mice received a single dose of Imipenem (12.5 mg/kg; Alfa Aesar) following CLP. Mice were treated with metformin (65 mg/kg i.p.) once a day for four consecutive days, i.e. administered on 3, 4, 5, and 6 days after CLP.

### *P. aeruginosa*-induced pneumonia in mice

*Pseudomonas aeruginosa* deposition into the mouse pharynx followed by aspiration was conducted using previously described methods^30–32^. In brief, mice anesthetized with isoflurane were suspended by their upper incisors on a 60° incline board, tongue was gently extended followed by oropharyngeal deposition of PBS alone (control; 50 µl) or *P. aeruginosa*, wild-type strain K (PAK; 2.5 × 10^7^/mouse) in PBS (50 µl). Lung homogenates were prepared four hours after PAK instillation and serial dilutions used to determine colony-forming units (CFUs/ml) on agar plates.

### Reagents and antibodies

Metformin, RPMI 1640, and LPS were from Sigma-Aldrich (St. Louis, MO). 5-Aminoimidazole-4-carboxamide-1-β-d-ribofuranoside (AICAR) was purchased from Enzo (Farmingdale, NY). AMPK activators MK8722 and A769662 were from MedChemExpress (Monmouth Junction, NJ). Antibodies: phospho-Thr172-AMPK, AMPKα1, phospho-Ser93-beclin1, phospho-Ser79-Acetyl CoA Carboxylase, and LC3B were obtained from Cell Signaling Technology (Beverly, MA). Parkin and β-actin were from Santa Cruz Biotechnology (Dallas, TX), whereas anti-T1α IgG from R&D Systems (Minneapolis, MN). Horse Radish Peroxidase-conjugated antibodies were obtained from Bio-Rad (Hercules, CA). Emulsion oil solution containing 4′,6-diamidino-2-phenylindole (DAPI) from Vector Laboratories (Burlingame, CA). Hoechst dye from Life Technologies (Grand Island, NY) was applied to visualized nuclei in live macrophages.

### Lung histology and imaging

Lung isolation and indirect immunofluorescence staining were performed as previously conducted in our laboratory^24,29,33–35^. Mouse lungs were inflated with 1 ml paraformaldehyde in PBS (4%) and embedded with paraffin. Prior to staining, lung section (5-µm-thick) were deparaffinized in serial solutions of Citrisolv (Fisher Scientific, Pittsburgh, PA), isopropyl alcohol, and water, followed by antigen retrieval via steaming in 10 mM citric acid (pH 6.0) for 20 min and cooling for additional 20 min. Next, lung sections washed with PBS and blocked with BSA (3%) for 90 min were incubated with anti-Parkin and anti-T1α antibody overnight, at 4 °C. Secondary FITC or Rhodamine-labeled antibody were added for 60 min. Nuclei were stained using the emulsion oil solution containing DAPI. Fluorescence intensity was measured in randomly chosen areas of lung sections from control and post-sepsis mice, including mice that were treated with or without metformin. In selected experiments, fluorescent images were obtained from macrophages that were treated with Mitotracker Mito-SOX, LysoTracker, *E. coli* (Texas red conjugated) or macrophages that expressed GFP-LC3. Images were acquired using a confocal laser-scanning microscope Nikon A1R at the UAB High Resolution Imaging Facility. The levels of fluorescence in randomly selected areas were quantitated and displayed as two-dimensional scattergrams using HCImage, Hamamatsu’s image acquisition and analysis software. In selected experiments, the paraffin-embedded lung sections were processed with H&E staining.

### Macrophage and neutrophil isolation and culture

Mouse peritoneal neutrophils and macrophages were isolated as previously described^29,34–36^. Macrophages were elicited in 10- to 12-week-old mice by intraperitoneal application of Brewer thioglycollate (Sigma-Aldrich, B2551) and cells isolated 4 days after injection. Neutrophils were collected 4 h after thioglycollate injection. Cells were cultured in RPMI 1640 media supplemented with 8% FBS (Atlanta Biologicals, S11150), at 37 °C. For experiments where cytokines were measured neutrophils were used the same day of isolation, whereas macrophages were used after culture for 3–4 days prior to exposure to LPS.

### Conditioned media from LPS-treated macrophages

Peritoneal macrophages were treated with LPS (0 or 300 ng/ml) (Sigma-Aldrich, L2630) for 2 h. Cells were washed 2 times to remove LPS and then incubated in RPMI 1640 media supplemented with 8% FBS for an additional 24 h. Media were collected and used to culture alveolar epithelial cells for 24 h.

### Alveolar epithelial cell culture

L2, alveolar cell line (ATCC), cells were cultured in F-12K (Gibco, 21127022) supplemented with 8% FBS (Atlanta Biologicals, S11150) at 37 °C. In selected experiments, AECs were treated with conditioned media (RPMI 1640 media supplemented with 8% FBS) from unstimulated or LPS-treated macrophages.

### Bacterial killing assay ex vivo

*Park2*^+*/*+^ and *Park2*^*−/−*^ macrophages or neutrophils were cultured with AICAR (0 or 500 μM) for 60 min followed by incubation with *P. aeruginosa* (tenfold) for an additional 60 min. Macrophages were lysed using 0.1% Triton-X100 (final concentration) for 10 min. Serial dilution was then performed on the surviving bacteria and cultured overnight on agar plates. The CFUs were counted for bacterial killing determination, as previously performed in our laboratory^37^. In selected experiments, wild type and Parkin deficient peritoneal macrophages were treated with or without direct activators of AMPK, including MK8722 (0.6 µM) or A769662 (10 µM).

### Measurement of macrophage bioenergetics

The bioenergetics of macrophages was determined using the XFe96 analyzer from Seahorse Bioscience, which measures O_2_ consumption and proton production (pH) in intact cells, as performed previously^24,33,38^. The O_2_ consumption rate (OCR) is correlated with oxidative phosphorylation, and proton production (extracellular acidification rate) can be related to glycolysis. Measurements were performed using macrophages (1 × 10^5^) that were plated on XF96 plates, after which they were treated with AICAR (150 μM) for 48 h. The plate was then washed with XF assay buffer [DMEM, 5% FBS supplemented with 5.5 mm, d-glucose, 4 mm l-glutamine, and 1 mm pyruvate (pH 7.4)] and incubated in XF buffer for 30–60 min before the assay. After the assay, the cells were lysed with radioimmune precipitation assay (RIPA) buffer, and protein concentration was determined by Bradford assay. All results were corrected to protein levels in individual wells.

### Cytokine ELISA

ELISA was used to measure cytokine levels in culture media and bronchoalveolar lavage (BAL) fluids as previously described^33,35^. Levels of TNF-α and MIP-2 were determined using ELISA kits according to manufacturer’s instructions by R&D Systems (Minneapolis, MN).

### Western blot analysis

Western blot analysis was performed as described previously^24,33^. Each experiment was carried out three or more times with cell populations obtained from separate groups of mice.

### Protein concentration and cell counts in BAL fluids

Briefly, protein concentration in BAL fluid was determined by Bradford assay with Bio-Rad protein assay dye reagent concentrate (Bio-Rad, 500-0006). Neutrophils in BAL fluids were determined after cytospin and Wright-Giemsa staining followed by image acquisition using light microscopy^27^.

### Statistical analysis

One-way ANOVA with Tukey’s post hoc test was used to determine the statistical significance among multi groups, with normal distribution. For two groups, statistical significance was established using Student’s *t*-test. These analyses are performed with Microsoft Excel and Prism GraphPad (version 8.4.2). A *P* value <0.05 is considered significant.

## Results

### Parkin undergoes degradation in monocytes of sepsis survivors and post-sepsis mice

**Figure 1:**
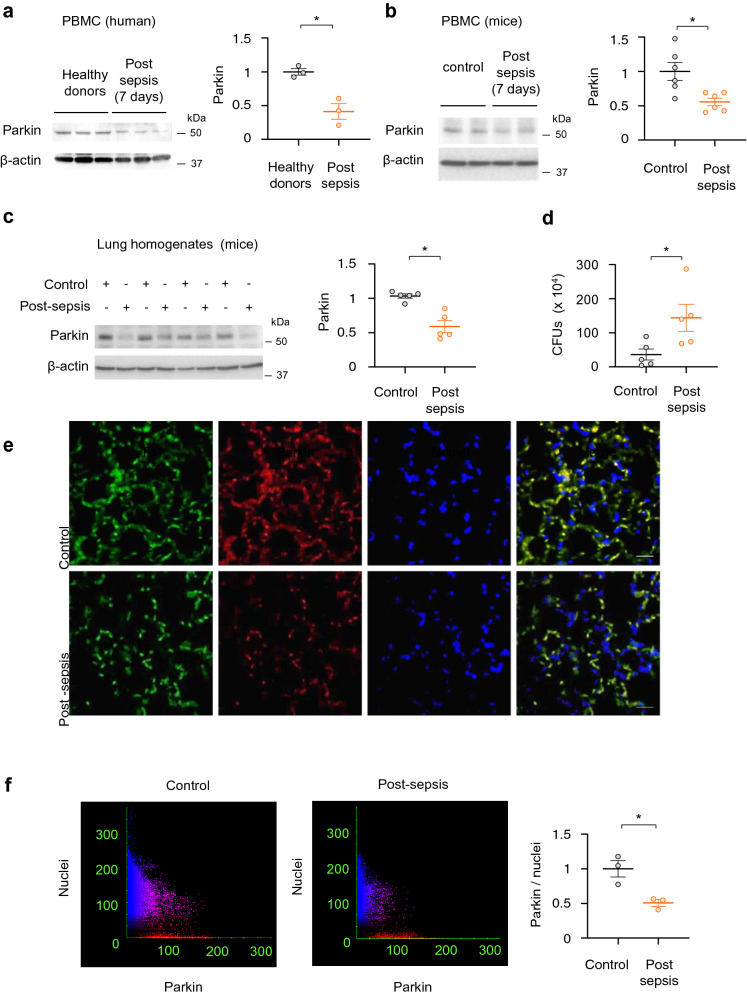
Parkin protein is diminished in leukocytes from sepsis survivors and post-sepsis mice. (**a**–**c**) Representative Western blots and quantitative analysis of Parkin in PBMCs from; (**a**) healthy donors or sepsis survivors, (**b**) control *vs.* post-septic mice, i.e. 7 days after CLP, and (**c**) whole lung homogenates in post-sepsis mice (24 h after CLP). Data presented as mean ± s.e.m., *n* = 3 (human) or *n* = 5–6 (mice). *P < 0.05, Student’s *t*-test. (**d**) The amount of bacteria (CFUs) in lung homogenates from control and post-CLP (7 days) mice. (**e**) Representative images show Parkin immunofluorescence in alveolar epithelial cells in lung sections from indicated groups of mice. T1-α (green), Parkin (red), nuclei (blue). Scale bar 100 µm. (**f**) Representative Scattergrams and quantitative analysis of Parkin/nuclei fluorescence intensity in lung sections of control and 24 h after CLP. Mean ± s.e.m., *n* = 3 mice/group. *P < 0.05, Student’s *t*-test.

The impact of microbial infections on Parkin protein level was determined in immune cells from healthy donors and patients that survived sepsis but developed immunosuppression. In peripheral blood mononuclear cells (PBMCs), significant decrease of Parkin was found 7 days after shock, as compared to control group (Fig. 1a). Similar to human PBMCs, Parkin is also diminished in leukocytes and whole lung homogenates obtained from mice 7 days after intra-abdominal polymicrobial sepsis (Fig. 1b,c). These mice were subjected to cecal ligation and puncture (CLP; see “Methods” section) that resulted in immune dysfunction and reduced capacity to kill *P. aeruginosa*, a virulent pathogen that causes secondary bacterial lung infections among patients that survive shock, (Fig. 1d)^39–41^. Type I alveolar epithelial cells (AECs) were immunostained using antibody for T1α, a mucin type transmembrane glycoprotein. Parkin degradation occurred 24 h post-CLP, as evidenced by Parkin immunofluorescence analysis of lung sections (Fig. 1e,f).

**Figure 2:**
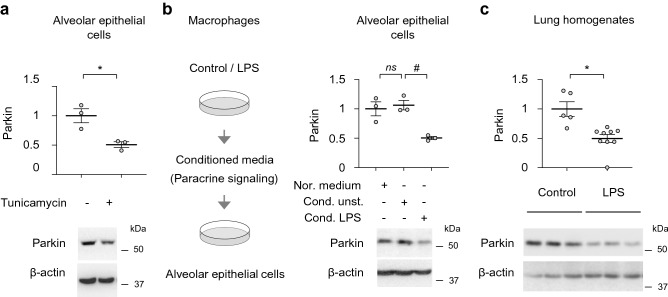
Parkin degradation in AECs is induced by ER stress and paracrine signaling from activated macrophages. (**a**) Representative Western Blots and optical band densitometry show Parkin protein levels in AECs Type I that were treated with Tunicamycin (0 or 10 μg/ml) for 24 h. (**b**) (*left*) Outline of experimental design to establish the impact of macrophage paracrine signaling on Parkin levels in AECs, and (*right*) representative Western blots and quantitative analysis of Parkin in AECs treated with or without conditioned media from control and LPS-treated macrophages. (**c**) Parkin levels in lung homogenates from control or mice treated with LPS (*i.t.*) for 24 h. (**a**–**c**) Data presented as mean ± s.e.m., (**a,b**) *n* = 3, and (**c**) *n* = 5–9. *P < 0.05, Student’s *t*-test; ^#^*P* < 0.05, *ns* not significant, ANOVA.

Subsequent experiments were focused on mechanism/s that are involved in Parkin degradation, including potential impact of endoplasmic reticulum (ER) stress, a relevant mechanism of AECs dysfunction in lung injury and fibrosis^15,42,43^. We found that Tunicamycin-mediated ER stress effectively triggered degradation of Parkin in AECs (Fig. 2a). Given that inflammatory conditions are linked to AECs injury, we also tested if macrophage-derived inflammatory paracrine signaling affects Parkin level in AECs. We found that conditioned media from LPS-activated macrophages stimulated Parkin degradation (Fig. 2b). Additional results confirmed that Parkin is diminished in lungs of mice subjected to LPS (*i.t.*) (Fig. 2c). These results indicate that proximal events associated with degradation of Parkin in AECs are associated with paracrine signaling mediated by activated macrophages and ER-stress. Importantly, reduced amounts of Parkin persisted in lungs and immune cells of post-sepsis mice, as well as in leukocytes from sepsis survivors.

### Parkin degradation in LPS-activated macrophages

**Figure 3:**
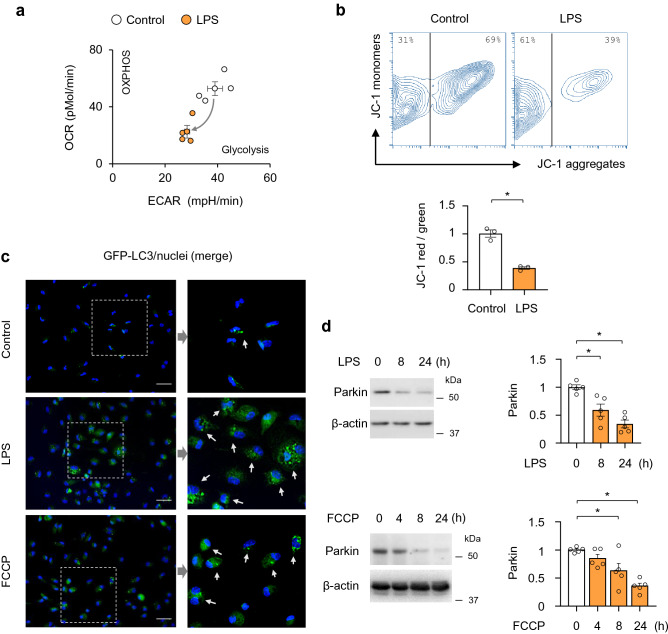
Parkin signaling and autophagy are engaged in activated macrophages. (**a**) Bioenergetic profile (OCR/ECAR) in macrophages-treated with LPS (0 or 30 ng/ml) for 24 h. Data presented as mean ± s.d., *n* = 5 individual measurements per group. (**b**) Flow cytometry contour plots and quantitative analysis of JC-1 red/green fluorescence in macrophages treated with or without LPS (30 ng/ml) for 30 min. Mean ± s.e.m., *n* = 3. *P < 0.05, Student’s *t*-test. (**c**) Representative images show accumulation of GFP-LC3 fluorescence in control, LPS or FCCP-treated macrophages for 4 h. Areas within dashed boxes are depicted in images on the right side. White arrows indicate cells positive for GFP-LC3 accumulation. GFP-LC3 (green), nuclei (blue). Scale bar 25 µM. (**d**) Western blot analysis of Parkin in macrophages treated with LPS (0 or 1 µg/ml) or FCCP (0 or 500 nM) for indicated time. Data presented as mean ± s.e.m., *n* = 5. *P < 0.05, ANOVA.

Previous studies have shown that mitochondria depolarization stimulated recruitment of Parkin and mitophagy^13,44,45^. Macrophage pro-inflammatory activation is typically associated with bioenergetic reprograming that affects oxidative phosphorylation (OXPHOS), however, both OXPHOS and glycolytic metabolism are decreased 24 h after exposure to LPS; evidence of decline in the macrophage bioenergetic capacity (Fig. 3a). Activation of Parkin signaling occurs as early as 30 min after LPS-treatment and dissipation of mitochondrial membrane potential (ΔΨ_m_). Dissipation of ΔΨ_m_ by LPS is determined using JC-1 probe, which accumulates in the polarized mitochondria and at the high concentration emits red fluorescence due to J-aggregates formations, whereas it becomes green at low concentration when ΔΨm is diminished (Fig. 3b). Notably, either mitochondria uncoupling with LPS or direct dissipation of ΔΨ_m_ by FCCP led to activation of autophagy, as evidenced by accumulation of the autophagy adaptor protein, GFP-LC3B (Fig. 3c). Parkin is degraded within 8 h of LPS or FCCP treatment, suggesting the requirement of Parkin recruitment for the formation of phagosomes (Fig. 3d). These results indicate that Parkin is an essential component of autophagy formation in activated macrophages. However, Parkin is subsequently degraded, which likely limits further activation of the Parkin-autophagy signaling axis.

### Parkin-dependent autophagy is required for effective killing of bacteria by macrophages ex vivo and in the lungs of mice infected with *P. aeruginosa*

**Figure 4:**
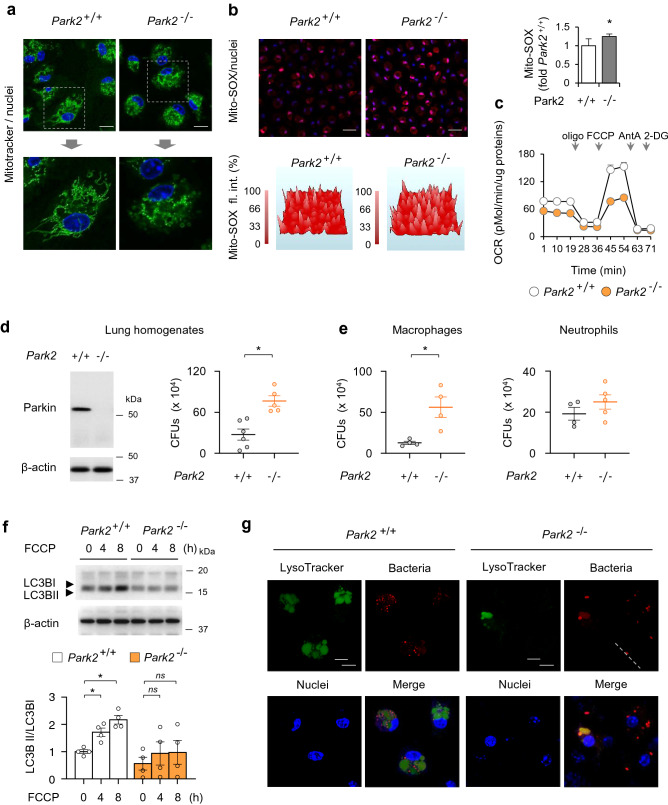
Parkin is required for phagolysosome formation and effective killing of bacteria. (**a**) Representative images show mitochondrial network in *Park2*^+*/*+^ and *Park2*^*−/−*^ macrophages. Mito-Tracker (green), nuclei (blue). Scale bar 10 µm. (**b**) Images, 3-D profiles, and Mito-SOX quantitation showing Mito-SOX fluorescence in *Park2*^+*/*+^ and *Park2*^*−/−*^ macrophages. Mito-SOX (red), nuclei (blue). Scale bar 50 µm. (**c**) Representative plot shows OCR indices (Mitochondrial Stress Test) in *Park2*^+*/*+^ and *Park2*^*−/−*^ macrophages. (**d**) Representative Western Blot (*left*) of Parkin in lungs of *Park2*^+*/*+^ and *Park2*^*−/−*^ mice. (*Right*) CFUs from lung homogenates of *Park2*^+*/*+^ and *Park2*^*−/−*^ mice that were subjected to bacteria (*i.t.*). Mean ± s.e.m., *n* = 5–6 mice per group. *P < 0.05, Student’s *t*-test. (**e**) CFUs after incubation of *Park2*^+*/*+^ and *Park2*^*−/−*^ macrophages or neutrophils with bacteria. Mean ± s.e.m., *n* = 4–5 individual measurements using cell populations obtained from 2 to 3 mice. *P < 0.05, Student’s *t*-test. (**f**) Western blot analysis of LC3BII/I in macrophages-treated with FCCP (0 or 500 nM) for indicated time. Mean ± s.e.m., *n* = 4. *P < 0.05, ANOVA. (**g**) Images depicted phagolysosome formation in *Park2*^+/+^ and *Park2*^−/−^ macrophages incubated with bacteria (red). LysoTracker (green), nuclei (blue). Scale bar 10 µm.

Parkin acquisition to depolarized mitochondria and mitophagy are well understood^13,44^, however, how Parkin contributes to macrophage host defense functions in sepsis and post-sepsis immunosuppression is less clear. In *Park2*^*−/−*^ macrophages, we found a significant decrease in the mitochondrial oxygen consumption rate (OCR) in association with the accumulation of fragmented and ROS-producing mitochondria (Fig. 4a–c). *Park2*^*−/−*^ macrophages are characterized by robust production of inflammatory cytokines, TNF-α and MIP-2, plus enhanced activation of the NLRP3 inflammasome (Fig. S1a,b), a major component of the pro-inflammatory response and potential indicator of mitochondrial dysfunction^46^. Previously, mitochondrial ROS have been shown to increase macrophages bacterial killing^47^. However, in spite of mitochondria depolarization and ROS flux, *Park2*^*−/−*^ mice have diminished capacity to kill *P. aeruginosa* (Fig. 4d,e). In particular, wild-type (*Park2*^+*/*+^) and Parkin deficient (*Park2*^*−/−*^) mice were subjected to pneumonia using *P. aeruginosa* strain K (PAK). Significant increases of colony-forming units (CFUs) indicated that *Park2*^*−/−*^ mice have diminished capacity to kill PAK, as compared to *Park2*^+*/*+^ mice (Fig. 4d). Infected mice showed accumulation of bacteria, immune cells flux, and cellular debris in alveolar spaces with thickened septae. These indices are more pronounced in Parkin deficient mice (Fig. S1c). Parkin deficient macrophages but not neutrophils also have reduced ability to kill PAK ex vivo (Fig. 4e), corroborating the observed deficiency in bacterial killing in lungs of *Park2*^−/−^ mice. These results indicate that Parkin deficiency confers an immunosuppressive phenotype, impaired bacterial killing, in mice. Because Parkin is diminished in immune cells of sepsis survivors (Fig. 1a), our findings also suggest that Parkin-deficiency is linked to risk of secondary bacterial lung infections.

Next, we determined whether loss of bacterial killing involves defective activation of autophagy. Indeed, the LC3BII/LC3BI ratios are increased after dissipation of ΔΨ_m_ in *Park2*^+*/*+^, but not in *Park2*^*−/−*^macrophages (Fig. 4f). Furthermore, *E. coli* effectively stimulates phagolysosome formation in *Park2*^+*/*+^ in contrast to *Park2*^*−/−*^ macrophages (Fig. 4g). These results suggest that Parkin-dependent autophagy, downstream of mitochondrial depolarization, is essential for effective killing of bacterial pathogens by macrophages.

### AMPK activates Parkin-independent autophagy and improves macrophage-mediated killing of bacteria

**Figure 5:**
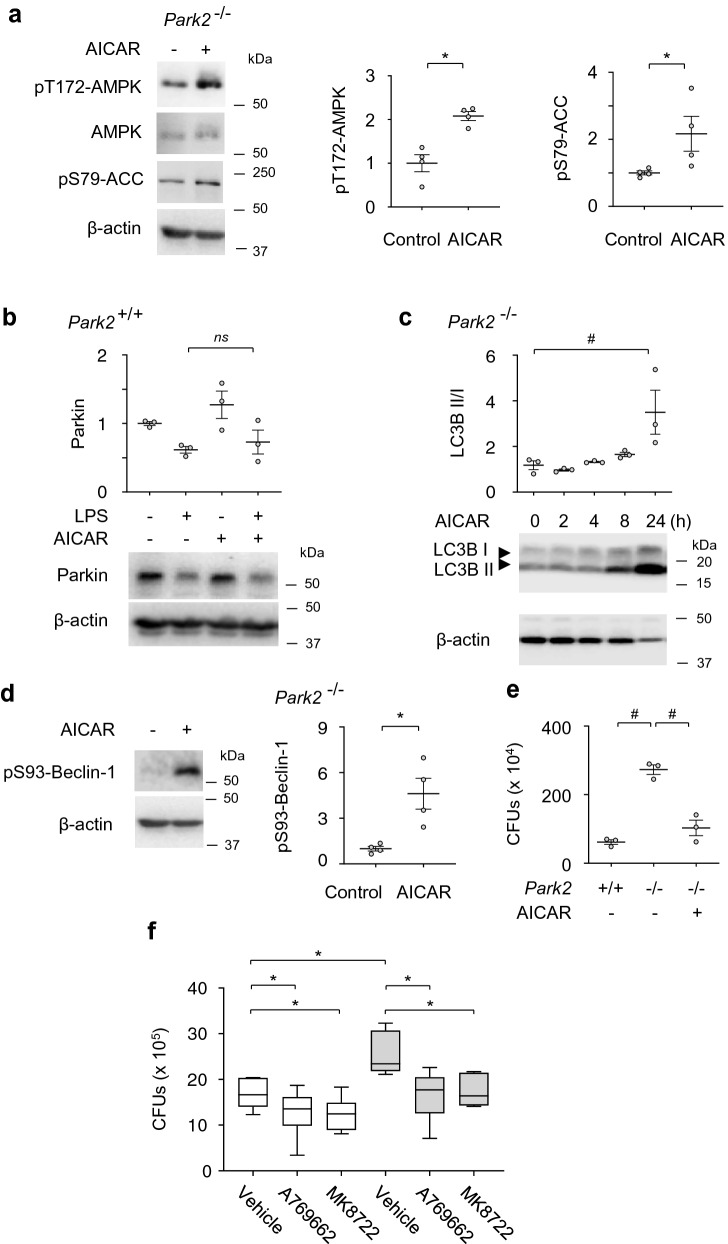
AMPK activation restored autophagy and bacterial killing in Parkin-deficient macrophages. (**a**) Western blot analysis of phospho AMPK and ACC in AICAR (0 or 500 µM) treated *Park2*^*−/−*^ macrophages, while (**b**) shows Parkin levels after sequential exposure to AICAR (0 or 500 µM) for 2.5 h and LPS (0 or 300 ng/ml) for 24 h. Mean ± s.e.m., *n* = 3–4. *P < 0.05, Student’s *t*-test; ^#^*P* < 0.05, ANOVA; *ns,* not significant. (**c**) LC3BII/I ratios in *Park2*^*−/−*^ macrophages-treated with AICAR (500 µM) for indicated time. Mean ± s.e.m., *n* = 3. *P < 0.05, ANOVA. (**d**) Representative Western Blots and optical bend densitometry of pS93-Beclin-1 in *Park2*^*−/−*^ macrophages-treated with or without AICAR (500 μM) for 4 h. Mean ± s.e.m., *n* = 4. *P < 0.05, Student’s *t*-test. (**e**) The amounts of CFUs after incubation of *Park2*^+*/*+^ and *Park2*^*−/−*^ macrophages with bacteria. *Park2*^*−/−*^ macrophages were also pre-treated with AICAR (0 or 500 µM). Mean ± s.e.m., *n* = 5. ^#^*P* < 0.05, ANOVA. (**f**) The effects of direct activators of AMPK on bacterial killing by peritoneal macrophages ex vivo. PAK CFUs are determined from Park2+/+ and Park2−/− macrophages that were treated with or without MK8722 (0.6 µM) or A769662 (10 µM) for 60 min prior to exposure to bacteria. Data are presented as Box Plot, n = 8–10. *P < 0.05, ANOVA.

AMPK is a master bioenergetic sensor and metabolic regulator, including mitochondrial function and autophagy^21,48,49^. Although Parkin and AMPK are major regulators of autophagy/mitophagy, crosstalk between these key metabolic regulators during sepsis and post-sepsis immunosuppression remains to be determined. Similar to metformin, AICAR (an AMP mimetic) induces activation (phosphorylation Thr-172) of AMPK (Figs. 5a, S2). However, it fails to preserve Parkin levels in LPS-stimulated macrophages (Fig. 5b). Although these results indicate that AMPK had no effect on Parkin degradation, we tested if AMPK activation promotes autophagy regardless of Parkin deficiency. In *Park2*^*−/−*^ macrophages, AICAR-dependent activation of AMPK effectively increased the LC3BII/LC3BI ratio, indicating autophagy activation (Fig. 5c). AMPK stimulates autophagy by direct phosphorylation of Beclin-1 and ULK1, or indirectly, via inhibition of the mTOR signaling pathway^22–24^. We confirmed that AMPK-induced autophagy involves phosphorylation of Ser93-Beclin-1, an early mediator of autophagy initiation (Fig. 5d). These findings indicate that AMPK activation-mediated autophagy can occur independently from Parkin recruitment. Next, we tested the functional effects of Parkin-independent AMPK activation on bacterial killing. AICAR significantly improved bacterial eradication in *Park2*^*−/−*^ macrophages, as demonstrated by reduced amounts of PAK CFUs (Fig. 5e). While AICAR is an excellent AMPK activator, the specific effects mediated by AMPK was also tested using direct AMPK activators MK8722 and A769662. Treatment of peritoneal macrophages with either A-769662 or MK8722 significantly diminished PAK CFUs, as compared to control (vehicle) (Fig. 5f). Similar decreases were obtained after exposure *Park2*^*−/−*^ macrophages to A-769662 or MK8722. These results indicate that the enhancement of bacterial killing by macrophages can be achieved using both indirect and direct activators of AMPK.

In addition to enhanced bacterial killing, AICAR improved mitochondrial bioenergetics in *Park2*^*−/−*^ macrophages, including reserve capacity and maximal OCR (Fig. S3a–g). Major components of the electron transport chain complexes I–IV, and ATP-synthase alpha subunit Complex V were increased in AICAR-treated *Park2*^−/−^ macrophages (Fig. S3h,i). These results show that AMPK activation enhanced *Park2*^−/−^ macrophage mitochondrial bioenergetics.

We also determined if AMPK affects lung injury in *Park2*^*−/−*^ mice. Exposure to LPS (2 mg/kg; i.t.) resulted in accumulation of immune cells and cellular debris in alveolar spaces (Fig. S4a). Lung injury is characterized by increased permeability (i.e. increased BAL fluid proteins), neutrophil accumulation, and production of inflammatory cytokines, TNF-α and MIP-2 (Fig. S4b–e). Importantly, indices of lung injury are reduced in mice treated with metformin *versus* vehicle. These findings show that AMPK activation diminishes severity of lung injury in Parkin knockout mice.

### The AMPK-Beclin-1 signaling axis improved bacterial killing in lungs of sepsis-immunosuppressed mice

**Figure 6:**
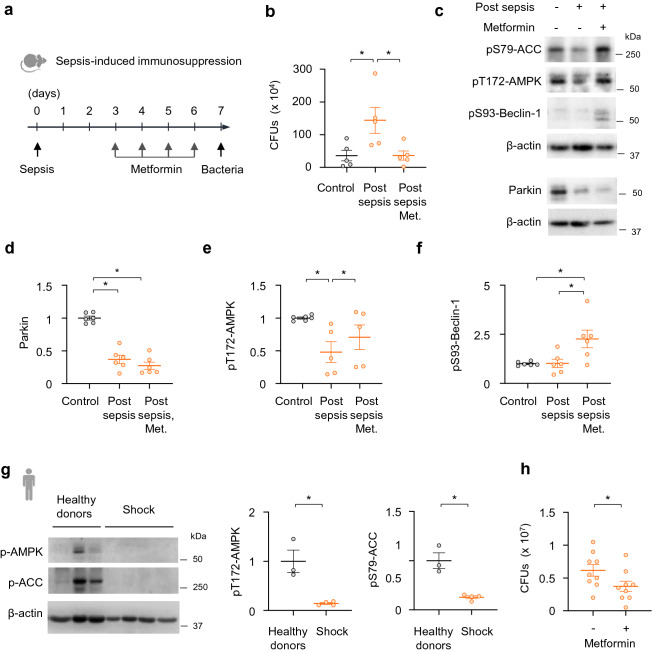
AMPK activator metformin activates Parkin-independent autophagy and improves bacterial killing in lungs of sepsis-immunosuppressed mice. (**a**) Outline of therapeutic dosing for metformin in sepsis-immunosuppressed mice followed by bacterial pneumonia. (**b**) CFUs from lung homogenates of indicated groups of mice. Mean ± s.e.m., *n* = 5 per control, post-sepsis and post-sepsis mice treated with metformin (Met.) groups. *P < 0.05, ANOVA. (**c**) Representative Western blots and quantitative analysis of (**d**) Parkin, (**e**) p-AMPK, and (**f**) pSer93-Beclin-1 from lung homogenates of control and sepsis-immunosuppressed mice. Data presented as mean ± s.e.m., *n* = 5–6. *P < 0.05, ANOVA*.* (**g**) Western blot analysis of pThr172-AMPK and pSer79-ACC and β-actin in monocytes from healthy donors and patients that survive shock (24 h after shock). Mean ± s.e.m., *n* = 3 (healthy donors), *n* = 4 (post-shock). *P < 0.05, Student’s *t*-test. (**h**) CFUs obtained after monocytes (shock) were incubated with or without metformin (1 mM) for 2.5 h. Mean ± s.e.m., *n* = 3 (patients) with three technical repetitions. *P < 0.05, Student’s *t*-test.

**Figure 7:**
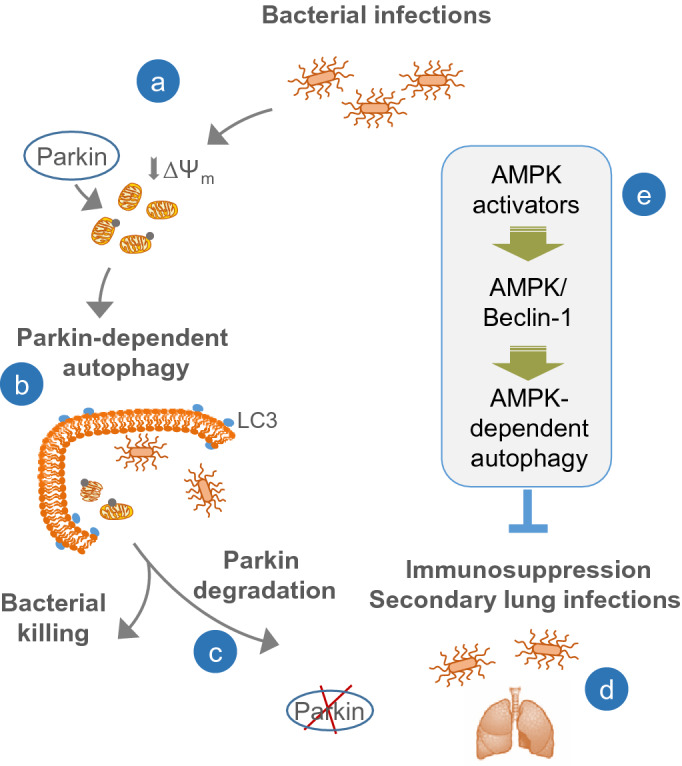
AMPK activates Parkin-independent autophagy in macrophages /monocytes and reduces severity of secondary bacterial lung infections. (**a**) Bacterial infections promote mitochondrial membrane depolarization (ΔΨ_m_) followed by Parkin recruitment and autophagy activation. (**b**) Parkin-dependent autophagy facilitates bacterial killing and resolution, (**c**) however; subsequent degradation of Parkin and loss of Parkin signaling is associated with (**d**) macrophage immunosuppressive phenotype and high risk of secondary lung bacterial infections. (**e**) AMPK-dependent autophagy reduces risk of secondary bacterial lung infections and respiratory failure.

Previous studies, including our own, have shown benefits of pre-emptive administration of AMPK activators in sepsis, including recent studies related to autophagy and mitochondrial biogenesis impairment in age-dependent liver injury, or cardiovascular complications in experimental models^50,51^. However, we applied a different strategy, in particular to use pro-survival models of sepsis in order to study the therapeutic (post-sepsis) effects of metformin on immunosuppression. This is a distinct concept compared to previous studies that utilized pre-emptive application of AMPK activators in lethal models of sepsis. To determine if AMPK activation restores the capacity for bacterial killing in lungs of post-sepsis mice, metformin (65 mg/kg; *i.p*.) was administered for four consecutive days during the immunosuppressive phase, as evidenced by increased bacterial number 7 days after CLP (Fig. 6a,b). Importantly, metformin significantly enhanced killing of *P. aeruginosa*, as compared to sepsis-immunosuppressed mice that received vehicle (Fig. 6b). AMPK activation had no effect on Parkin degradation in lungs of immunosuppressed mice (Fig. 6c,d); however, metformin efficiently activated the AMPK-Beclin-1 signaling axis (Fig. 6c,e,f). Notably, we also tested if metformin can improve bacterial killing by leukocytes obtained from shock survivors. While AMPK and acetyl CoA Carboxylase (ACC) phosphorylation are significantly diminished in leukocytes of post-shock patients (24 h), exposure to AMPK activator metformin (1 mM) for 4 h greatly improved MSSA killing ex vivo (Fig. 6g,h). These results indicate that AMPK can activate Parkin-independent autophagy in the lungs of post-sepsis mice and may also reduce susceptibility to secondary bacterial lung infections among sepsis survivors (Fig. 7).

## Discussion

Our data supports that, in the host response to bacterial infections, Parkin recruitment to depolarized mitochondria is essential for effective activation of autophagy and killing of bacterial pathogens by macrophages. Our results underscore the importance of Parkin and AMPK deficiency in immunosuppression which compromises innate immunity in lungs of post-septic mice, simulating the clinical context of immune dysfunction in sepsis survivors and individuals with PD. Besides immune dysfunction, Parkin deficiency has adverse effects on mitochondrial turnover in epithelial cells, which is implicated in lung cancer, chronic obstructive pulmonary disease and pulmonary fibrosis, conditions also known to have increased susceptibility to bacterial infections^14,15^. While Parkin deficiency has been previously linked to impaired clearance of *M. tuberculosis*^52,53^, whether this mechanism is more broadly relevant to clinical immunosuppression has not been previously demonstrated. Given its potential bacterial ancestry, mitochondria when not properly cleared, are capable of releasing harmful inflammatory mediators, including DAMPs and alarmins^54^. Notably, most recent studies have shown that Parkin degradation in macrophages and dendritic cells can trigger mitochondrial antigen presentation and the development of neuroinflammation^55,56^. Such neuroinflammation may account for the cognitive impairment associated with sepsis. Importantly, while we show that diminished/absent Parkin signaling can be overcome by pharmacologic activation of AMPK-dependent autophagy in macrophages, such findings may be relevant to other cell populations. For example, lung epithelial dysfunction is linked to a defect in Pink1/Parkin and impaired mitochondrial quality control, found in idiopathic pulmonary fibrosis and experimental models of lung fibrosis^15,57^. Similar issues can be found in other aging-related diseases^58,59^. In this context, our results suggest that activation of AMPK is an important therapeutic strategy, indeed, we have recently published that metformin-dependent activation of AMPK accelerates the resolution of lung fibrosis^24^. Interestingly, recent studies revealed AMPK and ATG1-induced lipophagy are required for DUOX activation and bacterial eradication upon enteric infection^60^. These results, and our previous findings, support protective actions of AMPK activation in acute lung injury and PD^25,61^.

AICAR, an analog of AMP, is widely used as an activator of AMPK, although selected studies have shown its limited specificity, including a potential impact on intracellular AMP/ATP ratio^62^. However, we have demonstrated that two direct activators of AMPK MK8722 or A-769662 also increased bacterial killing by macrophages, even upon deficiency of Parkin. MK8722 is a pan-AMPK activator that has been shown to effectively enhance activity of all 12 mammalian AMPK complexes (recombinant complexes) in vitro and improved insulin-independent glucose uptake along with glycogen synthesis, with improved glucose homeostasis in rodents and rhesus monkeys^63^. A-769662 has been shown to activate AMPK both allosterically and by inhibiting de-phosphorylation of AMPK on Thr-172. The impact of A-769662 to activate AMPK was evidenced by activation of AMPK that harbors a mutation in the AMPKγ subunit that abolishes activation by AMP^64^.

Metformin, prescribed for more than 40 million people with T2D worldwide, has been shown to reduce the severity of sepsis in preclinical and clinical retrospective studies^29,65^, although the mechanisms have remained elusive. Collectively, our findings support the concept that Parkin plays dual roles in mitochondrial quality control and host defense functions, supporting an evolutionarily conserved mechanism resulting from the proposed origin of eukaryotic mitochondria from bacteria.

## Supplementary Information

Supplementary Information.
