# Supplementary material for: AMPK activates Parkin independent autophagy and improves post sepsis immune defense against secondary bacterial lung infections
Source: Sci Rep. 2021 Jun 11;11:12387. doi: 10.1038/s41598-021-90573-0 (PMC8196038; doi:10.1038/s41598-021-90573-0)
Supplement: Supplementary file 1 — Supplementary Information. [file 41598_2021_90573_MOESM1_ESM.docx]

**AMPK Activates Parkin Independent Autophagy and Improves Post Sepsis Immune Defense against Secondary Bacterial Lung Infections**

Nathaniel B. Bone, Eugene J. Becker Jr., Maroof Husain, Shaoning Jiang, Anna A. Zmijewska, Dae-Won Park, Balu Chacko, Victor Darley-Usmar, Murielle Grégoire, Jean-Marc Tadie, Victor J. Thannickal, and Jaroslaw W. Zmijewski.

**Supplementary Figure 1**


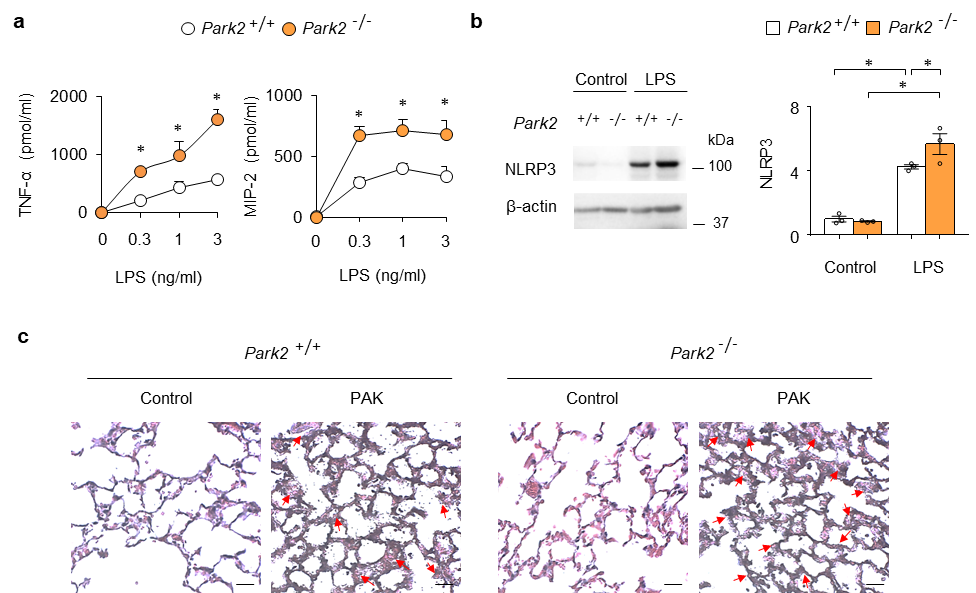


**Figure S1. Parkin deficient macrophages have enhanced production of pro-inflammatory cytokines and NLRP3 activation.** (**a**) TNF-α and MIP-2 levels in culture media of *Park2^+/+^* and *Park2^-/-^* macrophages treated with or without LPS for 4 hours. Data presented as mean ± s.d., *n* = 5. **P* < 0.05 (ANOVA). (**b**) NLRP3 levels in macrophages (*Park2^+/+^* and *Park2^-/-^*) treated with or without LPS for 16 hours. Representative western blots of NLRP3 and β-actin are shown. Mean ± s.e.m., *n* = 3. **P* < 0.05, (ANOVA). (**c**) Representative images (H&E) show lung sections from control and mice subjected to intratracheal instillation of *P. aeruginosa* strain K (PAK). Red arrows indicate areas of injury, including alveolar spaces filled with bacteria, cell infiltrates, cellular debris and thickened septae of PAK-treated mice. Scale bar, 500 µm.

**Supplementary Figure S2**


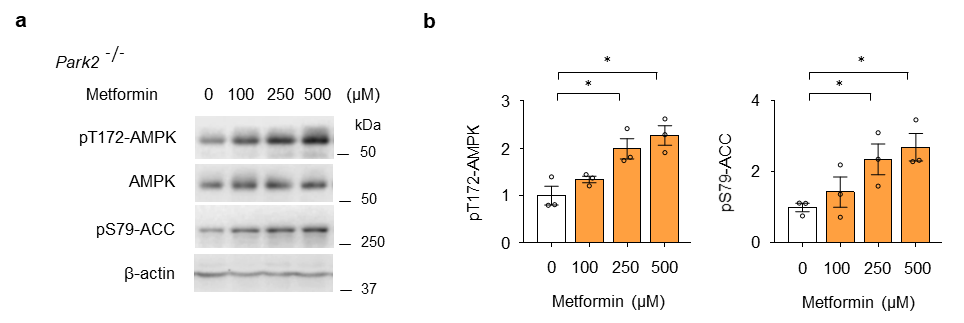


**Figure S2. Metformin-dependent activation of AMPK in Parkin deficient macrophages**. (**a**) Representative western blots and (**b**) optical densitometry of pT172-AMPK, AMPK and pS79-ACC in *Park2^-/-^* macrophages treated with metformin (0-500 µM) for 2.5 hours. Data presented as mean ± s.e.m., *n* = 3. **P* < 0.05 (ANOVA).

**Supplementary Figure 3**


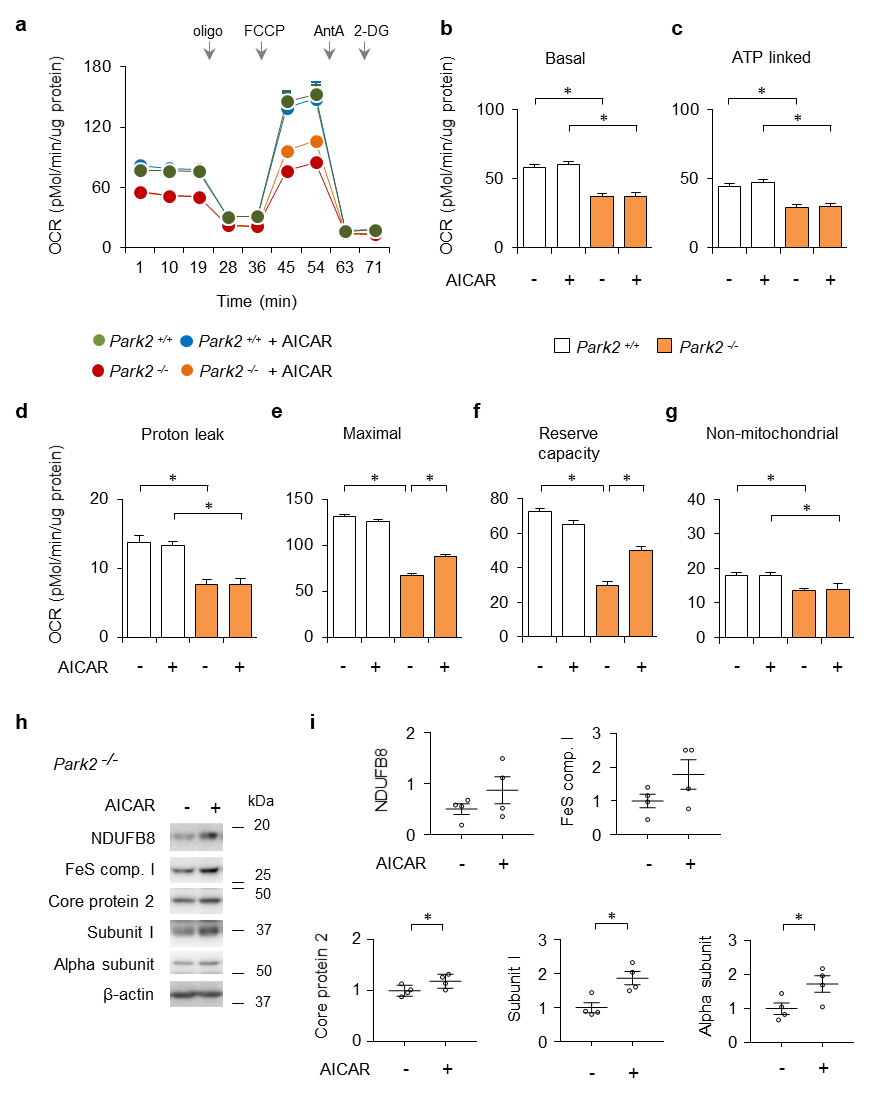


**Figure S3.** **The effects of AMPK activation on mitochondrial oxygen consumption rate (OCR) and biogenesis**. (**a**) Representative traces of OCR in *Park2^+/+^* and *Park2^-/-^* macrophages treated with or without AICAR (250 µM) for 24 hours. Data presented as mean ± s.d., *n* = 5. (**b**-**g**) Mitochondrial basal OCR, ATP linked, maximal, reserve capacity, proton leak and non-mitochondrial OCR are shown. Mean ± s.d., *n* = 4-5. **P* < 0.05 (ANOVA). (**h**-**i**,) Western blot analysis of selected proteins in mitochondrial electron transport chain subunits: complex I: NDUFB8, complex II: FeS comp. l, complex III: core protein 2, complex IV: subunit I, and complex V: alpha subunit. *Park2^-/-^* macrophages were incubated with AICAR (0 or 250 μM) for 72 hours. Mean ± s.e.m., *n* = 4, **P* < 0.05 (Student’s *t*-test).

**Supplementary Figure 4**


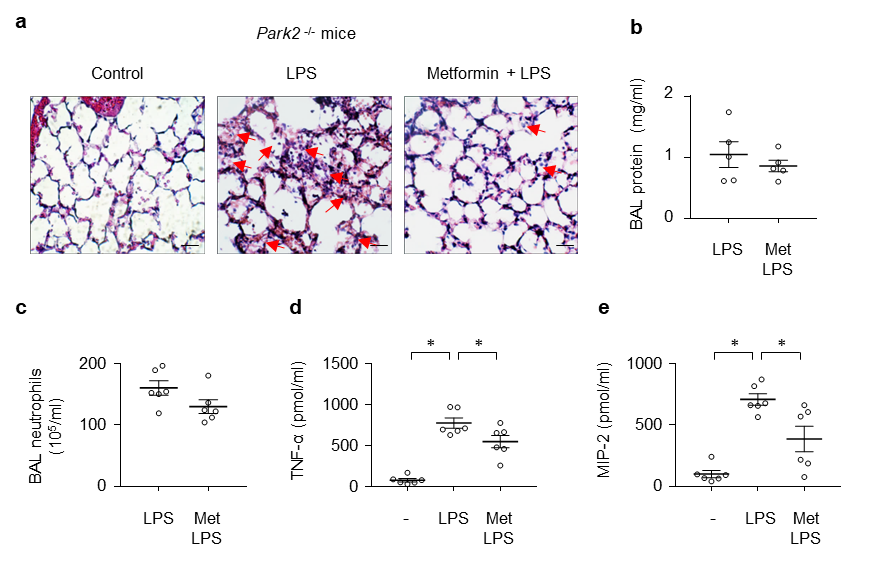


**Figure S4.** **Metformin reduces severity of endotoxin-induced ALI in *Park2^-/-^* mice**. (**a**) Representative images show H&E staining of lung sections from *Park2^-/-^* treated as indicated. Red arrows indicate spaces filled with a mixed mononuclear/neutrophilic infiltrate, cellular debris and proteinaceous material. Scale bar, 600 µm. (**b**-**e**) BAL analysis for neutrophil infiltrates, protein levels and pro-inflammatory cytokines, TNF-α and MIP-2, in indicated groups of *Park2^-/-^* mice. Mean ± s.e.m., *n* = 5-6. ^#^*P* < 0.05 (Student’s *t*-test) or **P* < 0.05 (ANOVA).

IMMUNOBLOTS

*Park2*

LPS

Control

+/+

-/-

+/+

-/-

LPS

Control

+/+

-/-

+/+

-/-

LPS

Control

+/+

-/-

+/+

-/-

Supplementary Figure 1b


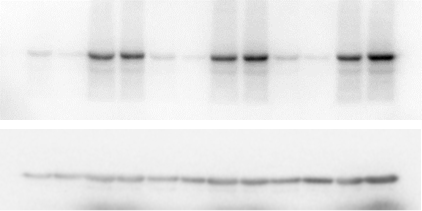


NLRP3

β-actin

Supplementary Figure 2a

M

0 100 250 500

0 100 250 500

0 100 250 500

(μM)

*Park2*

*-/-*

AICAR


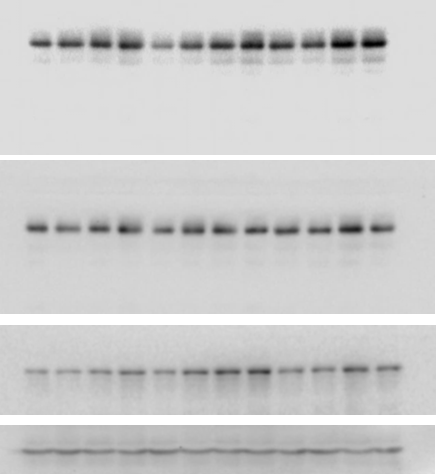


β-actin

p-ACC

AMPK

p-AMPK

Supplementary Figure 3h

Short exposure

Middle exposure

Long exposure

-

AICAR

-

-

+

+

-

-

-

-

+

+

-

-

AICAR

-

-

+

+

-

-

-

-

+

+

-

-

AICAR

-

-

+

+

-

-

-

-

+

+

-

MM

M

complex IV: subunit I

complex III: core protein 2

complex V: alpha subunit

complex II: FeS comp. l

complex I: NDUFB8

β-actin


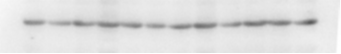


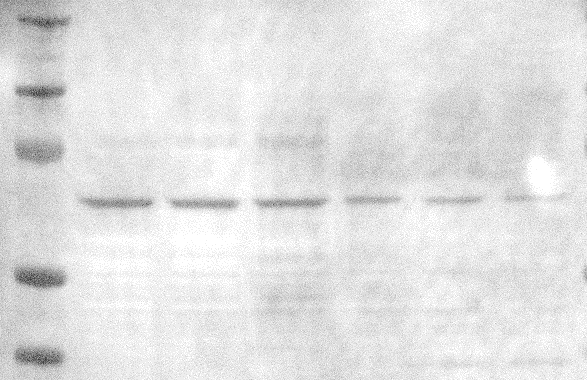

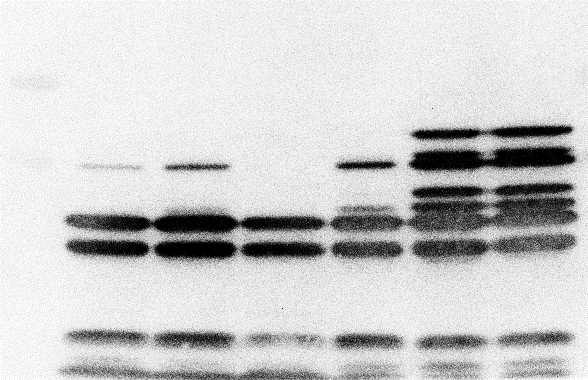

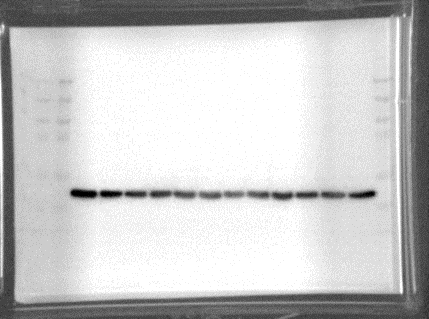

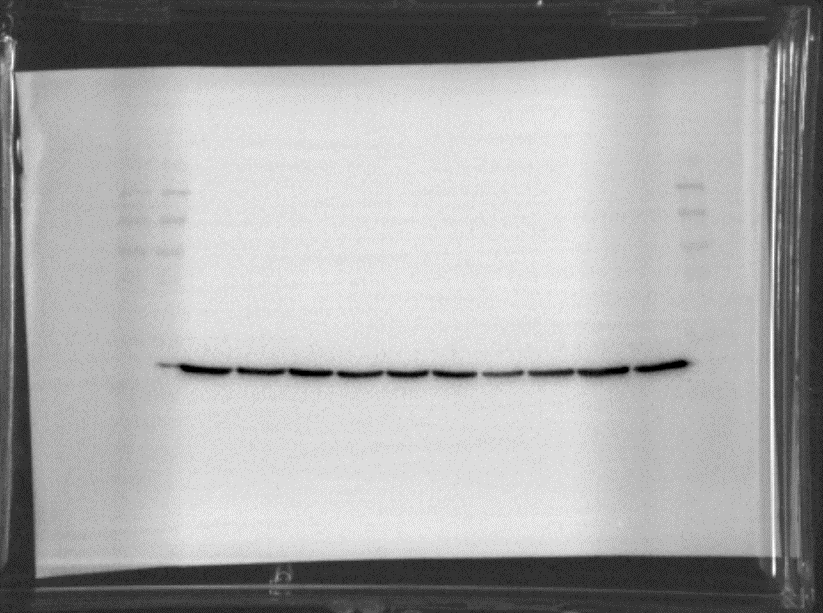

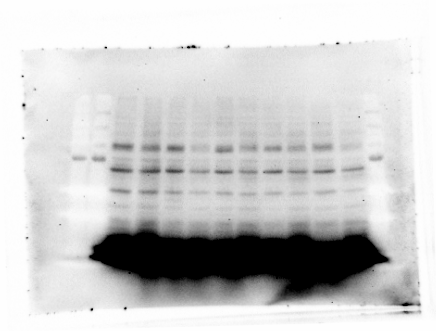


Fig. 1b

Fig. 1a

M

M

β-actin

M

M

M

M

M

M

50

37

50

Parkin

β-actin

50

Post

sepsis

(7 days)

Healthy

donors

Post sepsis

Control

M

M

+

+

+

+

+

+

75

M

M

M

Post-sepsis

Control

+

+

+

+

+

+

+

+

+

+

Parkin

37

Fig. 1c

Parkin

β-actin


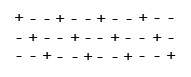

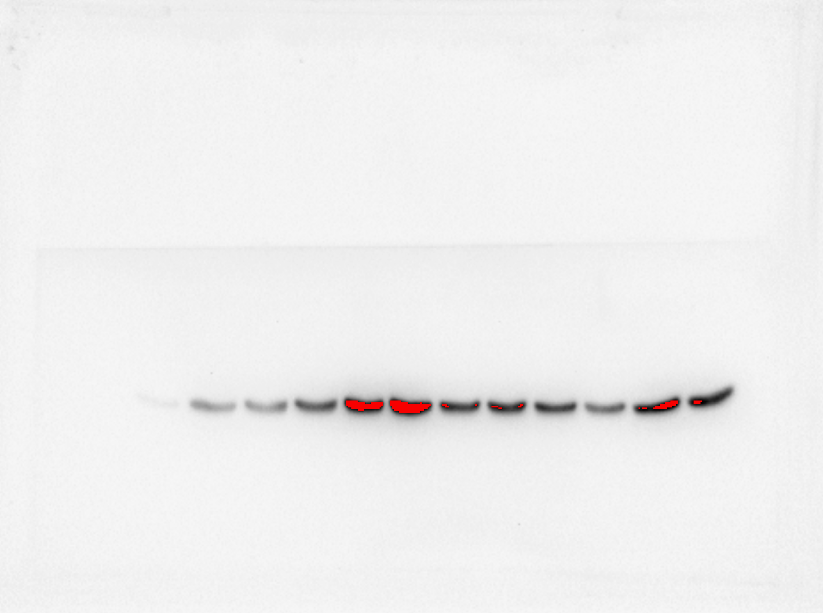

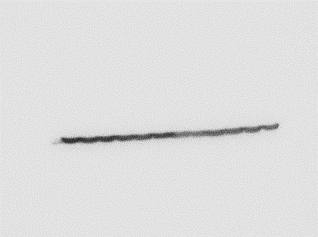

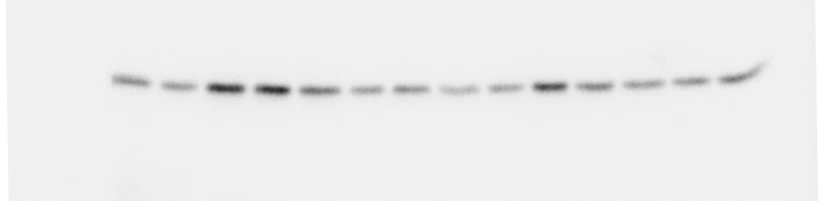

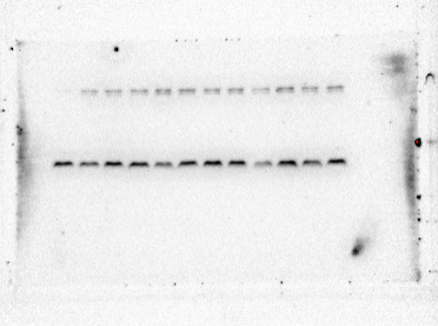

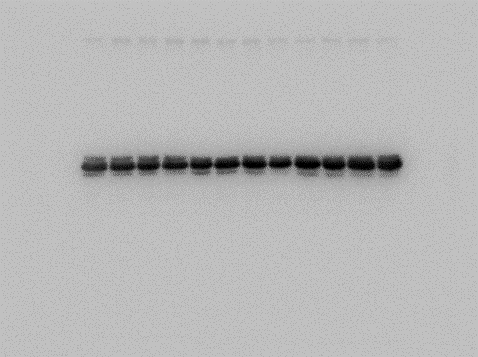

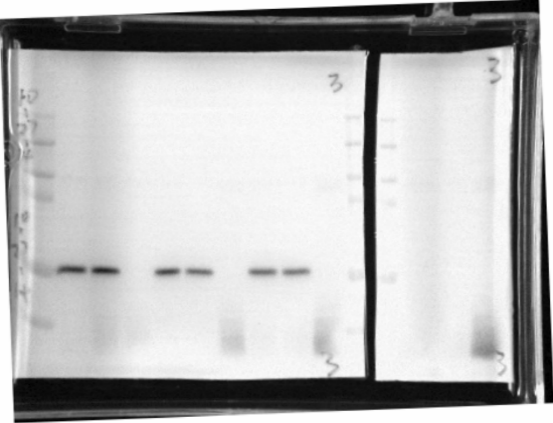

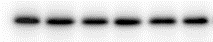


Fig. 2a

Fig. 2b

Fig. 2c

-

Nor. medium

Cond. unst.

Cond. LPS

CTL

LPS

CTL

LPS


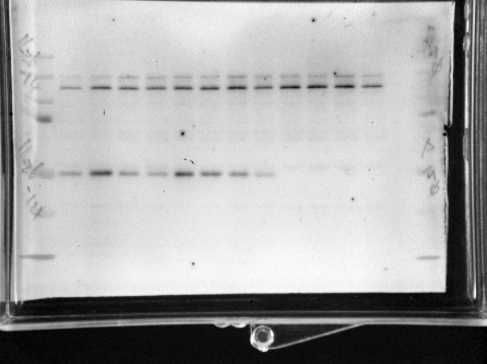

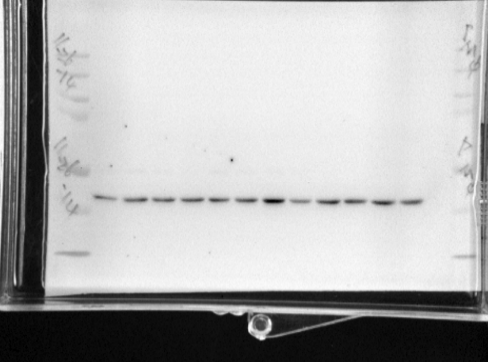


Fig. 4d

0

8

24

LPS

0

48

24

8

72

72

96

96

M

M

FCCP

0

4

8

24

(h)

0

4

8

24

4

8

24

0

M

M

M

*Park2*


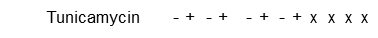


Parkin

Parkin

Parkin


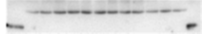


β-actin

MM

β-actin

β-actin

Fig. 3d

x

(h)

Parkin

Parkin

β-actin

β-actin

Fig. 4f


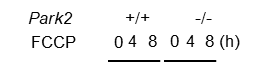


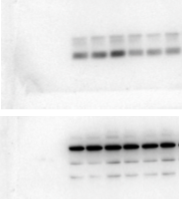

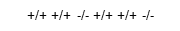


LC3BII

LC3BI

β-actin

Parkin

β-actin


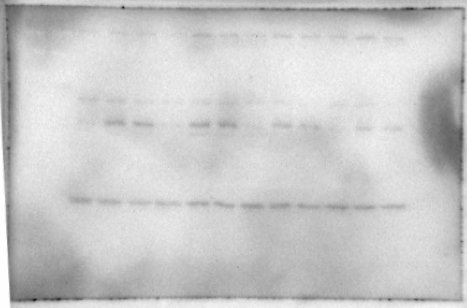

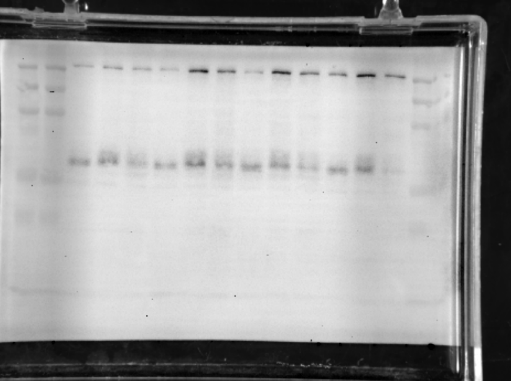

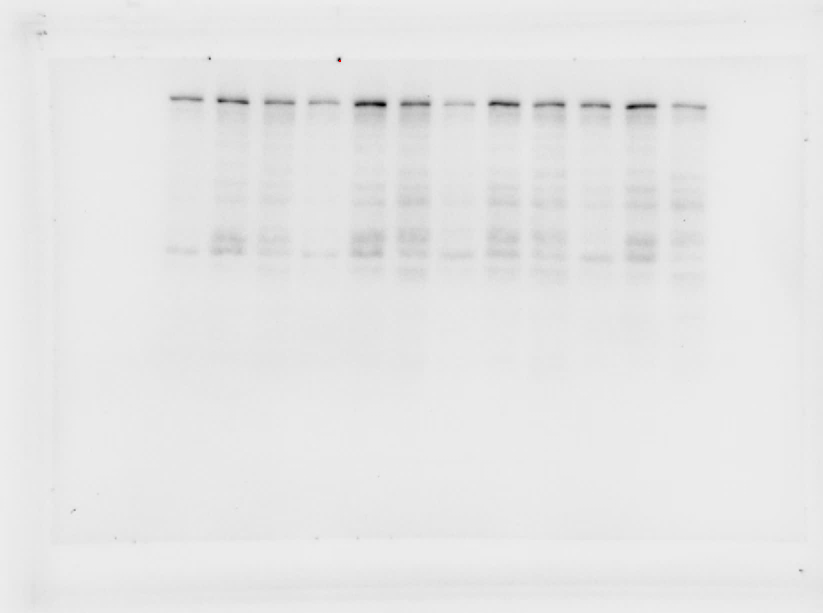

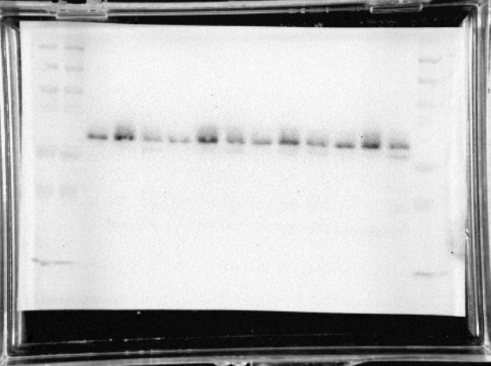

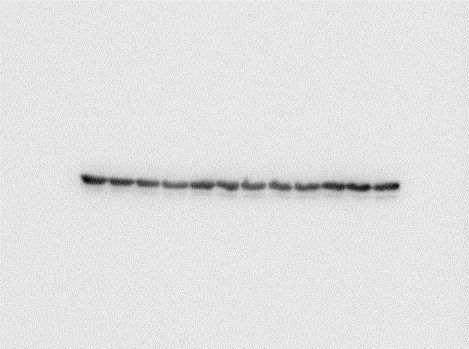

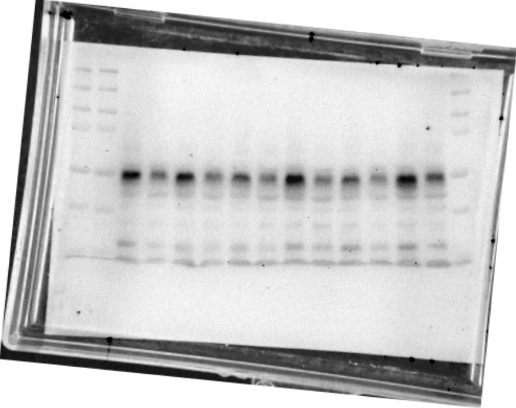

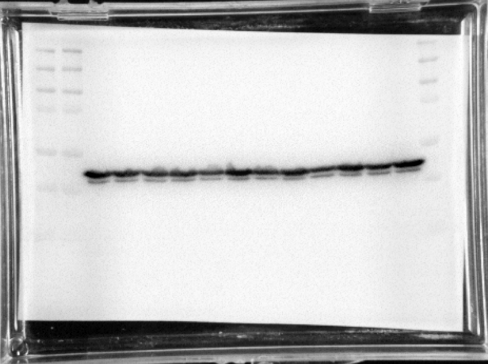

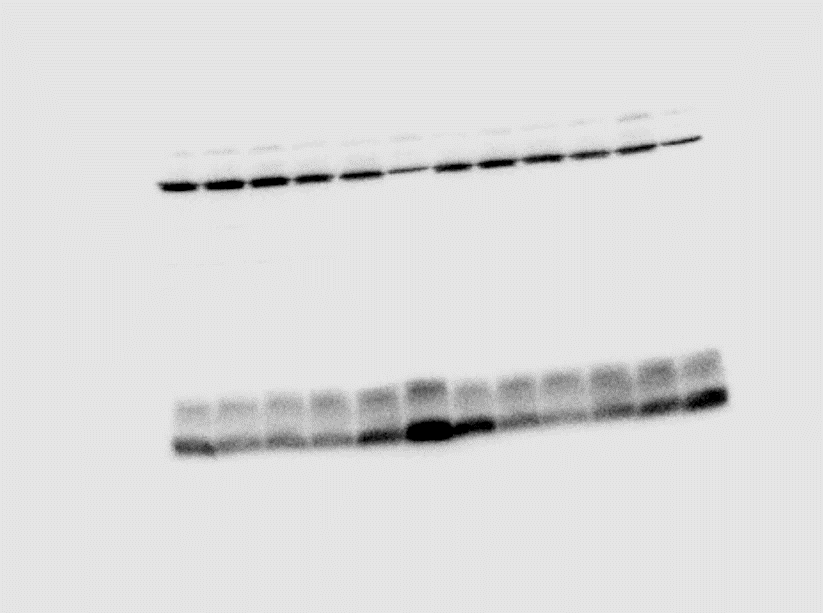

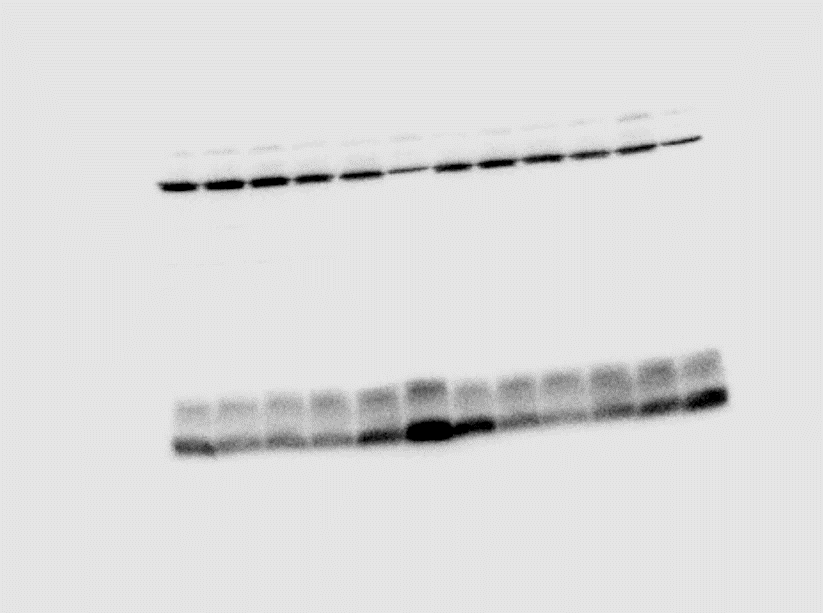


Fig. 5b

Fig. 5a

Fig. 5d

β-actin

t-AMPK

p-ACC

p-AMPK

p-Beclin-1

Parkin

β-actin

M

β-actin


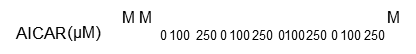

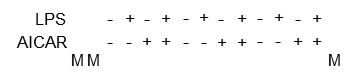


Fig. 5c


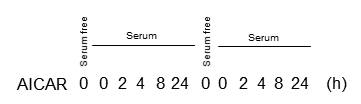


LC3BI

LC3BII

β-actin


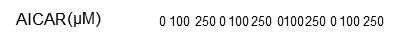


M

M


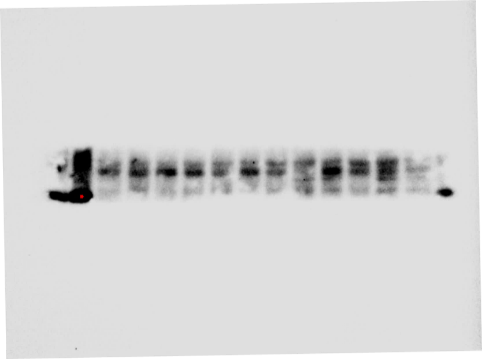

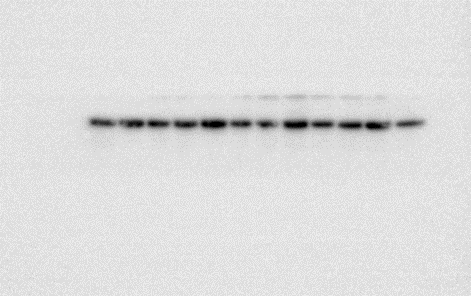


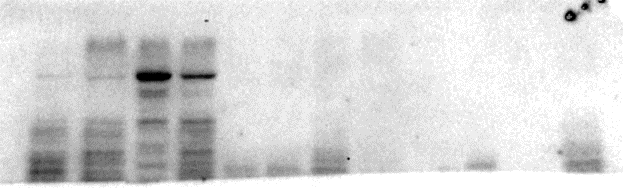

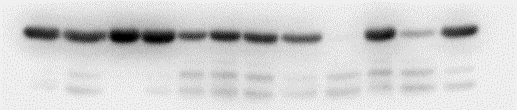

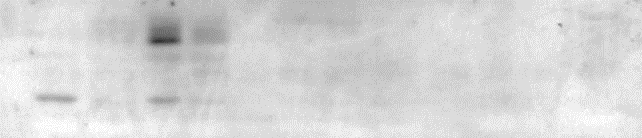


Fig. 6g

Fig. 6c

Healthy

donors

Shock

p-AMPK

p-ACC

β-actin


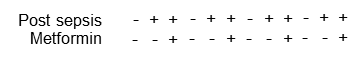


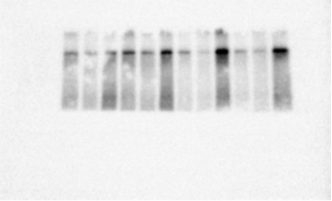


p-ACC

p-AMPK


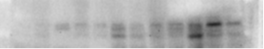


p-Beclin-1

β-actin


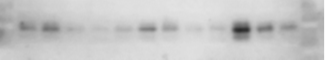


Parkin
